# Ultrastructure of the bacteriome and bacterial symbionts in the Asian citrus psyllid, *Diaphorina citri*

**DOI:** 10.1128/spectrum.02249-23

**Published:** 2023-12-04

**Authors:** Atsushi Nakabachi, Toshinobu Suzaki

**Affiliations:** 1 Research Center for Agrotechnology and Biotechnology, Toyohashi University of Technology, Toyohashi, Aichi, Japan; 2 Graduate School of Science, Kobe University, Kobe, Hyogo, Japan; University of Valencia, Valencia, Spain

**Keywords:** transmission electron microscopy, *Diaphorina citri*, bacteriome, *Carsonella*, *Profftella*, *Wolbachia*

## Abstract

The Asian citrus psyllid *Diaphorina citri* (Hemiptera: Psyllidae) is a notorious agricultural pest that transmits “*Candidatus* Liberibacter” spp. (Alphaproteobacteria: *Rhizobiales*), the pathogens of the destructive citrus disease huanglongbing. *D. citri* possesses a specialized organ called the bacteriome, consisting of uninucleate bacteriocytes and a syncytium. The bacteriocytes harbor “*Candidatus* Carsonella ruddii” (Gammaproteobacteria: *Oceanospirillales*), a nutritional symbiont that provides essential amino acids scarce in the phloem sap diet. The syncytium contains “*Candidatus* Profftella armatura” (Gammaproteobacteria: *Burkholderiales*), a defensive symbiont synthesizing diaphorin, a polyketide inhibitory to various organisms. Besides these obligate mutualists, *Wolbachia* (Alphaproteobacteria: *Rickettsiales*), a facultative symbiont, is also widespread in *D. citri*. To understand the morphological features of the bacteriome and symbionts, this study analyzed their ultrastructure using transmission electron microscopy. Putative *Wolbachia* cells were observed in the uninucleate bacteriocytes, which were packed with numerous *Carsonella* cells surrounded by host mitochondria, rough endoplasmic reticula, and the Golgi apparatus. *Carsonella* contained many ribosomes, parts of which formed conspicuous aggregates. The *Carsonella* envelope was lined with fiber bundles forming a net-like structure. In *Profftella*, numerous tubular structures were observed, each approximately 300 nm in diameter, consisting of several intertwined fibers. Some *Profftella* cells exhibited surface protrusions. The syncytium occasionally contained *Profftella* cells, apparently in the degradation process. Host organelles were less abundant in the syncytium than in the uninucleate bacteriocytes. Mitochondria in the syncytium had less developed cristae than those in the uninucleate bacteriocytes. These observations established a basis for understanding interactions among the host cells and bacterial symbionts.

**IMPORTANCE:**

Omics analyses suggested a mutually indispensable tripartite association among the host *D. citri* and organelle-like bacteriome associates, *Carsonella* and *Profftella*, which are vertically transmitted through host generations. This relationship is based on the metabolic complementarity among these organisms, which is partly enabled by horizontal gene transfer between partners. However, little was known about the fine morphology of the symbionts and the bacteriome, the interface among these organisms. As a first step to address this issue, the present study performed transmission electron microscopy, which revealed previously unrecognized ultrastructures, including aggregations of ribosomes in *Carsonella*, numerous tubes and occasional protrusions of *Profftella*, apparently degrading *Profftella*, and host organelles with different abundance and morphology in distinct cell types. These findings provide insights into the behaviors of the symbionts and host cells to maintain the symbiotic relationship in *D. citri.*

## INTRODUCTION

Various insect lineages possess a specialized organ called the bacteriome to harbor microbial symbionts (1 – 3). These insects are mainly exemplified by taxa that feed only on nutrient-deficient diets, including plant sap and vertebrate blood, and thus include numerous agricultural and medical pests. Although not only the phylogeny of symbionts but also the morphology, localization, and developmental origin of the bacteriome vary depending on insect lineages, bacteriome-associated symbionts tend to converge to organelle-like mutualists that provide essential nutrients for host survival (1 – 3). The Asian citrus psyllid, *Diaphorina citri* Kuwayama (Hemiptera: Sternorrhyncha: Psylloidea: Psyllidae: Diaphorininae), is a notorious agricultural pest that transmits “*Candidatus* Liberibacter” spp. (Alphaproteobacteria*: Rhizobiales*, hereafter *Liberibacter*), the pathogens of a destructive and incurable citrus disease known as huanglongbing or greening disease (4 – 6). While association with *Liberibacter* is transient, *D. citri* has more intimate and evolutionarily stable relationships with bacteriome-associated microbes that are transovarially transmitted through host generations (7 – 9). The abdominal hemocoel of *D. citri*, as in other psyllid species (10 – 16), contains a large yellow bilobed bacteriome that consists of numerous uninucleate bacteriocytes, a central syncytium surrounded by bacteriocytes, and an envelope to encase the whole organ (7 – 9). The uninucleate bacteriocytes harbor “*Candidatus* Carsonella ruddii” (Gammaproteobacteria: *Oceanospirillales*, hereafter *Carsonella*) (17), a typical nutritional symbiont, which provides the host psyllid with essential amino acids (8, 18 – 21) that are scarce in the phloem sap diet (22, 23). *Carsonella* is referred to as the “primary symbiont” because it is conserved among host species and is thus considered essential in Psylloidea (8, 17, 18, 21, 24 – 29). The syncytium of the psyllid bacteriome usually contains another bacterial lineage categorized as a “secondary symbiont,” which is phylogenetically diverse among psyllid lineages, suggesting its repeated replacements during the evolution of Psylloidea (19, 24 – 31). The secondary symbiont housed in the *D. citri* bacteriome is “*Candidatus* Profftella armatura” (Gammaproteobacteria: *Burkholderiales*, hereafter *Profftella*) (8, 9, 20, 26), a unique organelle-like defensive symbiont, whose primary role appears to protect the holobiont (host psyllid and bacteriome-associated mutualists) from natural enemies, using diaphorin, a polyketide inhibitory to various organisms (8, 20, 32 – 35). Omics analyses indicate a mutually indispensable tripartite association among the host *D. citri* and these symbionts. During their evolution as obligate mutualists confined in the bacteriome, the genomes of *Carsonella* and *Profftella* have been drastically reduced to 170 and 460 kb, respectively (8). *Carsonella* lacks numerous genes for biological processes apparently essential for survival but retains many genes for the biosynthesis of essential amino acids, which is consistent with the presumed role of *Carsonella* to supplement nutrition (8, 18 – 21). In contrast, *Profftella* has few genes for nutrient production, but 15% of the genome is devoted to gene clusters for the biosynthesis of diaphorin, a defensive secondary metabolite. The metabolic genes encoded in the *Profftella* genome are largely nonredundant with those of *Carsonella*, indicating complementarity between these symbionts (8). Furthermore, many host genes are upregulated in the bacteriome to complement the incomplete biosynthetic pathways deduced from the highly reduced *Carsonella* genome (16, 36). The majority of them are native animal genes, but some are shown to be of bacterial origin, which the host psyllid has horizontally acquired. Most notable are two divergent gene copies encoding argininosuccinate lyases that are responsible for the terminal step in the arginine biosynthesis pathway. Phylogenetic analyses strongly suggest that these genes are directly transferred from *Carsonella* (16). An ortholog is retained in the *Carsonella* genome but appears to have acquired a novel function independent of arginine biosynthesis. Another notable example is a gene (*ribC*) involved in the biosynthesis of riboflavin or vitamin B_2_ (16, 36), which is also scarce in the phloem sap diet (22). Although *Profftella* lacks most genes for nutritional provisioning, it exceptionally retains all genes for the riboflavin biosynthetic pathway other than *ribC* (8), suggesting metabolic complementarity between *Profftella* and the host psyllid (16). The donor bacterium of the psyllid *ribC* is uncertain but appears neither *Profftella* nor *Carsonella*. Although these horizontally acquired genes were first discovered in *Pachypsylla venusta* (Psylloidea: Carsidaridae), screening of genomic/transcriptomic data of *D. citri* and the potato psyllid *Bactericera cockerelli* (Triozidae) identified at least nine genes of bacterial origin shared by the three divergent psyllid species, indicating that the gene transfer events have occurred before the radiation of the major psyllid lineages (16). The mutually indispensable tripartite association based on genetic complementarity among the host and symbionts should depend on coordinated metabolic processes. However, little is known about how and which compounds (e.g., metabolites, proteins, or RNAs) are transferred among the host cytoplasm and symbionts.

In addition to these obligate mutualists, *Wolbachia* (Alphaproteobacteria: *Rickettsiales*), a facultative symbiont, has been detected in many *D. citri* populations in the world (7, 26, 37 – 44). Fluorescence *in situ* hybridization (FISH) analysis showed that *Wolbachia* resides in various types of *D. citri* cells, including uninucleate bacteriocytes (43). As with other hemipteran insects (1, 45 – 63), evidence is accumulating that interactions among the host psyllid, bacteriome-associated obligate mutualists, facultative symbionts, and plant pathogens are important for psyllid biology and host plant pathology (6, 8, 9, 15, 16, 18, 26 – 29, 34, 35, 41, 44, 64 – 66). Intriguing examples include a horizontal transfer of a transporter gene from the *Profftella* lineage to the *Liberibacter* lineage, indicating ecological and evolutionary interactions between the devastating plant pathogen and the bacteriome-confined mutualist (64). However, little is known about the fine morphology of the *D. citri* bacteriome and associated symbionts, which is essential for understanding cytological interactions among these organisms. Although limited numbers of transmission electron micrographs (TEM) of the *D. citri* bacteriome were previously reported (8, 67, 68), the information was minimal, only showing outlines of the organ. As a first step to address this issue, this paper reports the ultrastructure of the *D. citri* bacteriome and associated symbionts; obligate mutualists *Carsonella* and *Profftella*, and facultative *Wolbachia*.

## MATERIALS AND METHODS

### Insects

An established colony of *D. citri*, originally collected from the island of Amami Oshima, Kagoshima, Japan, was maintained on the saplings of the orange jasmine, *Murraya paniculata* (Rutaceae), individually covered with insect-rearing sleeves (L70 cm × W30 cm, Bugdorm Store; Taichung, Taiwan). The plant pots were kept in incubators at 28°C with a 16:8 (light:dark)-h photoperiod. Our previous microbiome analysis revealed that this colony is free from *Liberibacter* but is infected with *Wolbachia* of supergroup B, besides *Carsonella* and *Profftella* (26). Adults 5–10 days after emergence were collected from *M. paniculata* saplings using an insect aspirator and then caged in a plastic dish on ice for 5 min to immobilize them. They were sexed under a stereomicroscope, and bacteriomes were dissected from adult females of *D. citri* in phosphate-buffered saline.

### Transmission electron microscopy

The dissected bacteriomes were fixed with 2% paraformaldehyde and 2% glutaraldehyde in 0.1 M sodium phosphate buffer [0.1 M NaH_2_PO_4_ and 0.1 M Na_2_HPO_4_ (pH 7.4)] for approximately 24 h. After rinsing with 0.05 M potassium phosphate buffer [0.05 M KH_2_PO_4_ and 0.05 M Na_2_HPO_4_ (pH 7.4)], the specimens were postfixed with 2% osmium tetroxide in 0.05 M potassium phosphate buffer at 4°C for 2 h. After rinsing with 0.05 M potassium phosphate buffer, the fixed specimens were dehydrated in a graded series of ethanol (30%, 50%, 70%, and 90% once, and with 100% thrice, 15 min for each step). Subsequently, the specimens were treated with propylene oxide thrice for 10 min to replace ethanol, which were then embedded in Quetol 651 epoxy resin. The resin was polymerized at 60°C for 24 h. Ultrathin (80–90 nm thick) sections were obtained by ultramicrotomy and stained with 2% uranyl acetate and modified Sato’s lead solution [1.0 g each of Pb(NO_3_)_2_-3H_2_O, Pb(CH_3_COO)_2_-3H_2_O, and 2.0 g of Na_3_C_6_H_5_O_7_-2H_2_O dissolved in 82 mL of distilled water and 18 mL of 4% NaOH aqueous solution] (69). TEM observation was performed using a JEOL JEM-1200EX electron microscope or Hitachi H-7600.

## RESULTS

### Overview

**Fig 1:**
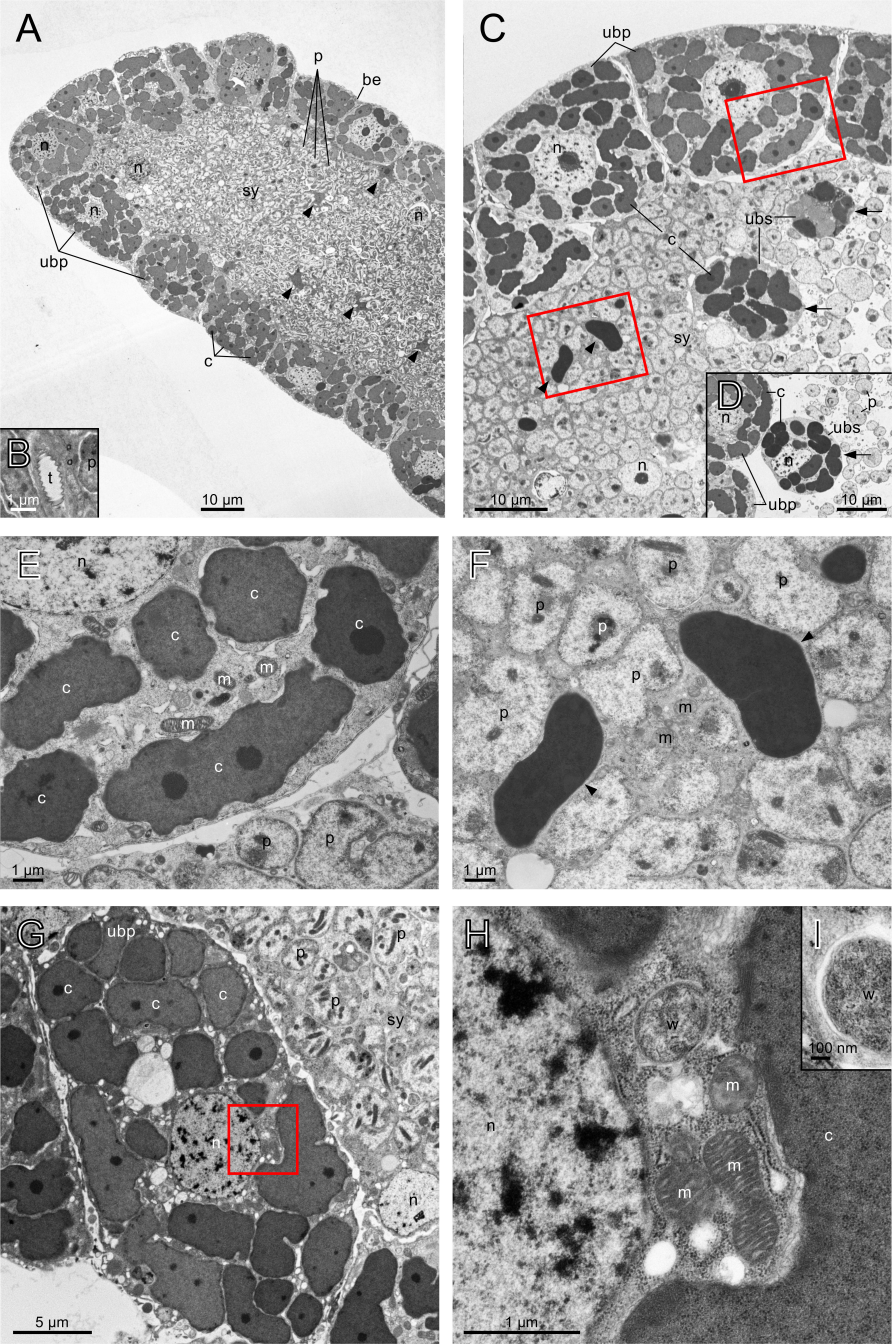
Transmission electron micrographs showing an overview of the bacteriome in adult females of *D. citri*. (**A**) The basic structure of the *D. citri* bacteriome. Uninucleate bacteriocytes harboring *Carsonella* surround the central syncytium harboring *Profftella*. Limited numbers of *Carsonella*-like structures (arrowheads) are observed in the syncytium. (**B**) Tracheole observed in the syncytium. (**C**) Uninucleate bacteriocytes (arrows) and *Carsonella*-like structures (arrowheads) observed in the syncytium. (**D**) Another example of the uninucleate bacteriocyte observed in the syncytium. (**E**) Enlarged image of the area in the upper rectangle in C. *Carsonella* cells are observed in a peripheral uninucleate bacteriocyte surrounding the syncytium. (**F**) Enlarged image of the area in the lower rectangle in C. *Carsonella*-like structures are observed along with *Profftella* in the syncytium. (**G**) A peripheral uninucleate bacteriocyte containing a structure that appears to be *Wolbachia*. (**H**) Enlarged image of the area in the rectangle in G. The putative *Wolbachia* cell is comparable in size to the transverse sections of mitochondria but lacks crista. (**I**) A putative *Wolbachia* cell with a gap between its double-layered cell envelope and the outermost structure, which appears to be the host membrane encasing *Wolbachia*. be, bacteriome envelope; c, *Carsonella*; m, mitochondrion; n, host nucleus; p, *Profftella*; sy, syncytium; t, tracheole; ubp, uninucleate bacteriocyte at the peripheral region; ubs, uninucleate bacteriocyte in the syncytium; w, putative *Wolbachia*.

The overall structure of the bacteriome of *D. citri* was as previously described (7 – 9). It consisted of numerous uninucleate bacteriocytes harboring *Carsonella*, a central syncytium harboring *Profftella*, and an envelope enclosing the whole organ (Fig. 1A). Some tracheoles were observed in the syncytium, indicating that this region is aerated (Fig. 1B). Although most uninucleate bacteriocytes were located peripherally to surround the central syncytium (Fig. 1A), the cells of the same type were occasionally observed within the syncytium (arrows in Fig. 1C and D). Moreover, a few structures resembling *Carsonella* in the bacteriocyte (Fig. 1E) were also observed in the syncytial cytoplasm (arrowheads in Fig. 1A, C, and F). As previous FISH analyses did not detect clear signals of free *Carsonella* cells in the syncytium (8, 9), further studies are required to determine whether these structures are truly *Carsonella*. Relatively small numbers of putative *Wolbachia* cells were observed in the uninucleate bacteriocytes (Fig. 1G through I) but not in the syncytium. The putative *Wolbachia* cells appeared mostly spherical, with a diameter of approximately 600 nm, much smaller than *Carsonella* and *Profftella*. They were comparable in size to the transverse sections of mitochondria but easily distinguishable as they lacked crista (Fig. 1H and I). The morphology of these cells was highly similar to that of TEM images of *Wolbachia* observed in various cell types of diverse host organisms, including the bacteriocyte of the whitefly *Bemisia tabaci* (Hemiptera: Sternorrhyncha: Aleyrodoidea: Aleyrodidae), the phloem of the cotton plant *Gossypium hirsutum* (Malvales: Malvaceae) that *B. tabaci* feeds on (70), ovary cells of the mosquito *Aedes aegypti* (Diptera: Culicidae) (71), cell lines of the mosquitos *A. aegypti* and *Anopheles gambiae* (Diptera: Culicidae) (72), and the fruit fly *Drosophila melanogaster* (Diptera: Drosophilidae) (73), and hindgut cells and midgut caeca of the woodlouse *Armadillidium vulgare* (Crustacea: Malacostraca: Isopoda) (74). Some of the putative *Wolbachia* cells in *D. citri* bacteriocytes showed a gap between the double-layered cell envelope and the outermost structure, which appeared to be the host membrane encasing *Wolbachia* (Fig. 1I).

### *Carsonella* and uninucleate bacteriocytes

**Fig 2:**
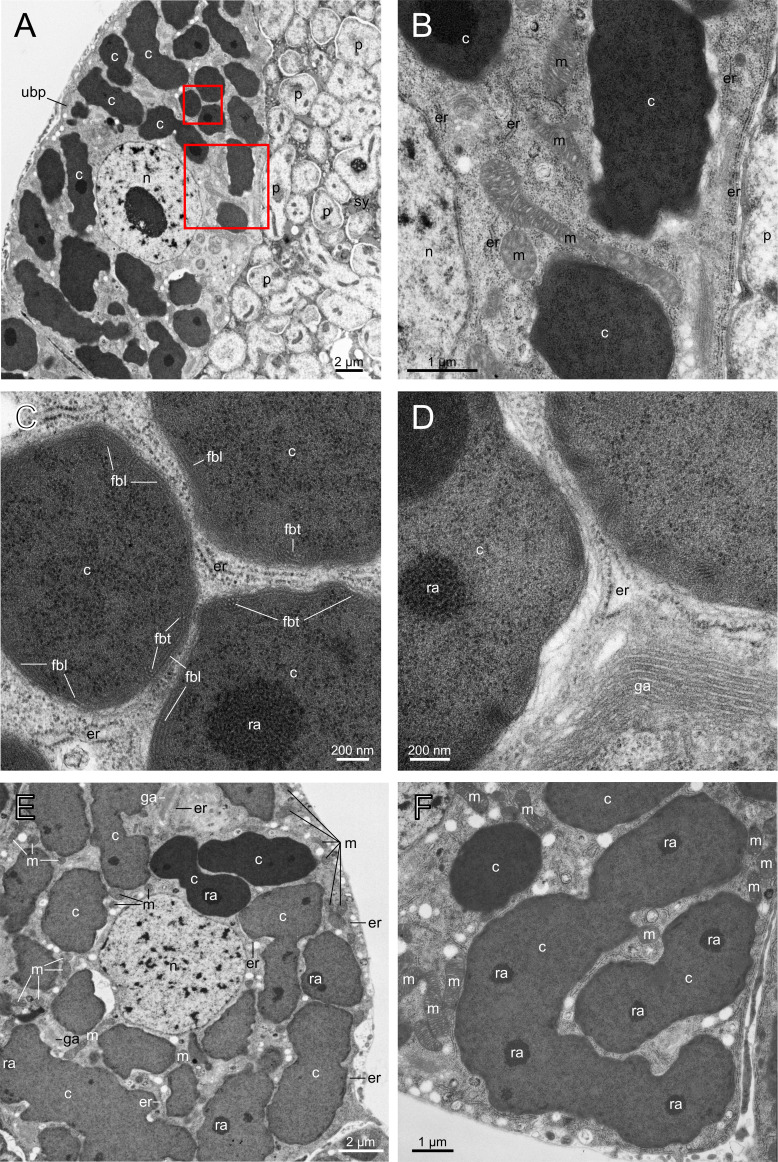
Ultrastructure of the uninucleate bacteriocyte and *Carsonella*. (**A**) Peripheral uninucleate bacteriocyte surrounding the syncytium. (**B**) Enlarged image of the area in the lower rectangle in A. *Carsonella* cells are adjacent to mitochondria and endoplasmic reticula with ribosomes. (**C**) Enlarged image of the area in the upper rectangle in A. *Carsonella* cells contain numerous ribosomes, some of which form conspicuous aggregates. Fiber bundles are observed near the surface of *Carsonella* cells. (**D**) *Carsonella*, a Golgi apparatus, and endoplasmic reticula with ribosomes adjacent to one another. (**E** and **F**) Other images of peripheral uninucleate bacteriocytes surrounding the syncytium. *Carsonella* cells show a close proximity with host organelles, including mitochondria, endoplasmic reticula with ribosomes, and the Golgi apparatus. A variation in electron density among *Carsonella* cells is observed. c, *Carsonella*; er, endoplasmic reticulum with ribosomes; fbl, fiber bundle arranged longitudinally; fbt, fiber bundle arranged transversely; ga, Golgi apparatus; m, mitochondrion; n, host nucleus; p, *Profftella*; ra, ribosomal aggregate; sy, syncytium; ubp, uninucleate bacteriocyte at the peripheral region.

Numerous sections of *Carsonella* were observed in the uninucleate bacteriocyte (Fig. 2A), which was consistent with previous DNA staining and FISH observations, showing that uninucleate bacteriocytes are packed with pleiomorphic, mostly tubular, *Carsonella* cells (9, 18, 21). The uninucleate bacteriocytes were rich in mitochondria ( Fig. 2A, B, E, and F), endoplasmic reticula with ribosomes (rough endoplasmic reticula, Fig. 2B through F), and the Golgi apparatus (Fig. 2D and E). Many mitochondria (Fig. 2A, B, E, and F) and rough endoplasmic reticula (Fig. 2B through F) were observed surrounding and in contact with *Carsonella* cells, implying close associations between *Carsonella* and these host organelles. Although less abundant, Golgi apparatuses were likewise observed in close proximity to *Carsonella* (Fig. 2D and E). While it is thought that *Carsonella* is enveloped in a triple-layered membrane (12, 13), the ultrastructure of the *Carsonella* envelope was not clearly observed in this study. However, fiber bundles were observed just under the envelope of *Carsonella* (Fig. 2C). These structures appeared to correspond to the “peripheral macula” previously reported for *Carsonella* of other psyllid species, *Cacopsylla pyricola* (Psyllidae: Psyllinae) (12) and *Anomoneura mori* (Psyllidae: Psyllinae) (13). The fiber bundles were arranged both longitudinally and transversely, suggesting to form a net-like structure lining the envelope of *Carsonella. Carsonella* cells were highly electron dense, and numerous darker spots were observed therein (Fig. 1 and 2). *Carsonella* contained a large number of ribosomes, which may partly account for the high electron density (Fig. 2B through D). The dark spots that previous studies referred to as “unidentified electron-dense aggregates” (17) or “central granular masses” (13) appeared to be aggregations of ribosomes (Fig. 2C and D). There was a remarkable variation in electron density among *Carsonella* cells, even within a single bacteriocyte (Fig. 1A, 2E and F). We currently have no idea what accounts for this variation.

### *Profftella* and syncytium

**Fig 3:**
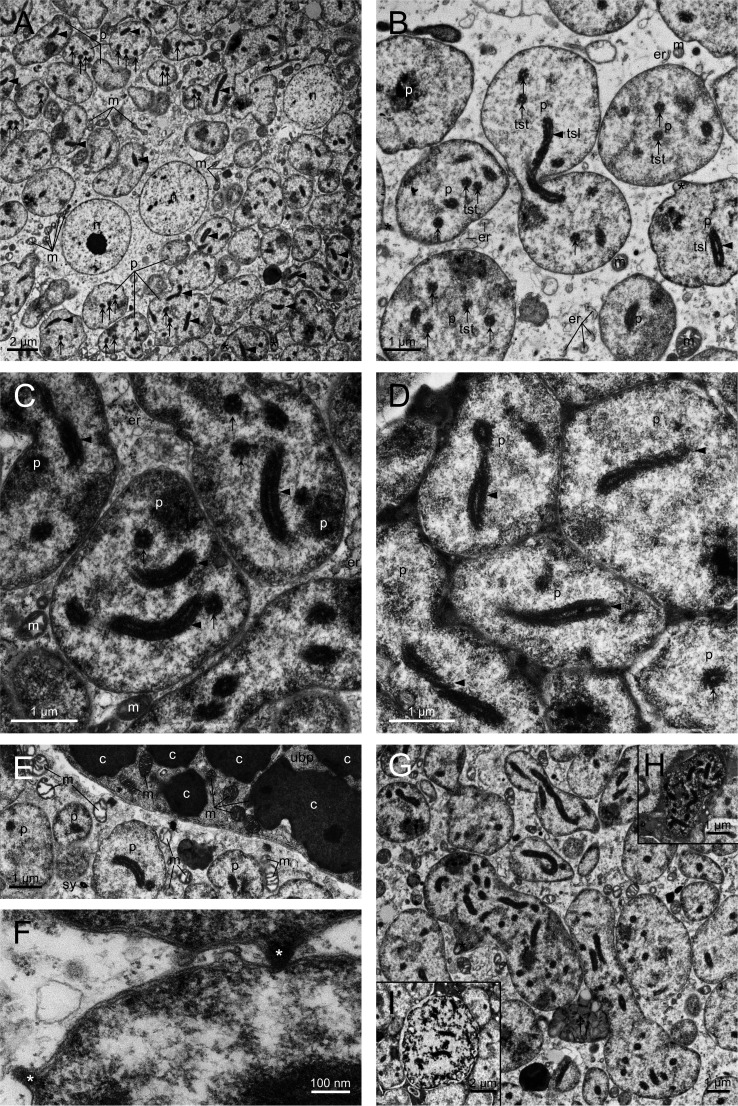
Ultrastructure of the syncytium and *Profftella*. (**A**) Syncytium containing *Profftella*, along with cell organelles including mitochondria. Tubular structures are observed in *Profftella*. Arrows and arrowheads indicate tubular structures sectioned transversely and longitudinally, respectively. Protrusions (asterisks) are observed in several *Profftella* cells. (**B**) *Profftella* cells containing tubular structures. Arrows and arrowheads indicate tubular structures arranged transversely and longitudinally, respectively. The asterisk indicates a protrusion from *Profftella*. Mitochondria and endoplasmic reticula with ribosomes are observed around *Profftella* cells. (**C**) Tubular structures observed in *Profftella* cells. Arrows and arrowheads indicate tubular structures sectioned transversely and longitudinally, respectively. (**D**) Tubular structures arranged longitudinally in *Profftella* cells. The structures appear to consist of intertwined fibers. (**E**) The interface of a uninucleate bacteriocyte harboring *Carsonell*a and the syncytium harboring *Profftella*. Mitochondria in the syncytium appear to have less developed cristae than their uninucleate bacteriocyte counterparts. (**F**) Three-layered envelopes that encase the *Profftella* cells. Protrusions (asterisks) are occasionally observed. (**G**) A structure that appears to be in the decomposition process (dagger), which was observed in the syncytium. The asterisk indicates a protrusion from *Profftella*. (**H**) and (**I**) Structures that appear to be *Profftella* cells in the decomposition process. c, *Carsonella*; er, endoplasmic reticulum with ribosomes; m, mitochondrion; n, host nucleus; p, *Profftella*; sy, syncytium; tsl, tubular structure sectioned longitudinally; tst, tubular structure sectioned transversely; ubp, uninucleate bacteriocyte at the peripheral region.

Numerous sections of *Profftella* with various shapes were observed in the syncytium (Fig. 3A through I), suggesting that *Profftella* are pleiomorphic. The *Profftella* cells were much less electron dense than *Carsonella* (Fig. 1 to 3). Conspicuous components found in *Profftella* cells were tubular structures sectioned both transversely (arrows in Fig. 3A through D) and longitudinally (arrowheads in Fig. 3A through D). These structures were approximately 300 nm in diameter, consisting of several intertwined fibers (Fig. 3C and D). It appeared that a single *Profftella* cell can contain multiple tubes. Although ribosomes were less abundant in *Profftella* than in *Carsonella*, many appeared to colocalize with the tubes (Fig. 3C and D). *Profftella* cells were observed to be encased in a three-layered envelope, the outermost of which probably corresponds to a host-derived membrane (Fig. 3F). Some *Profftella* cells exhibited protrusions on the surface (asterisks in Fig. 3A, B, F, and G). The syncytium occasionally contained structures that appeared to be *Profftella* cells in the degradation process (Fig. 3G through I). Host organelles, including mitochondria and endoplasmic reticula with ribosomes, were observed in the syncytium, but they were less abundant than in the uninucleate bacteriocytes (Fig. 3A through I). Furthermore, mitochondria in the syncytium had fewer cristae than those in the uninucleate bacteriocytes (Fig. 3E).

## DISCUSSION

This study revealed the ultrastructure of the bacteriome and associated symbionts in *D. citri*. The overall structure of the bacteriome was as previously described, in which most uninucleate bacteriocytes were located peripherally to surround the central syncytium (Fig. 1A). However, some uninucleate bacteriocytes were situated within the syncytium (Fig. 1C and D), which may facilitate the transport of compounds, potentially including metabolites, proteins, or RNAs, between *Carsonella* and *Profftella*. Furthermore, a few structures resembling *Carsonella* were also observed in the syncytial cytoplasm (Fig. 1A, C and F). Although previous FISH analysis detected freed *Carsonella* cells in the hemocoel of *D. citri* (9), which were likely in the process of transfer from the bacteriome to the ovary where the symbionts infect oocytes, no such cells were clearly observed in the syncytium. If the structures found in the present study are truly *Carsonella*, this proximity to *Profftella* may further facilitate material transfer between these symbionts. However, because the environmental conditions appear to differ between the uninucleate bacteriocyte and the syncytium, as suggested by the distinct abundance and morphology of host organelles (Fig. 2 and 3), the syncytium may not be a suitable habitat for *Carsonella*. Further studies are required to determine whether these structures are truly *Carsonella*. Besides, limited numbers of *Wolbachia* cells were observed in the uninucleate bacteriocytes (Fig. 1G, H and I), which potentially allow *Wolbachia* to enjoy the environment of the bacteriocyte, where various metabolites are assumed to be actively synthesized and transferred among the host and symbionts (16, 36). However, previous analyses showed that the relative abundance of *Wolbachia* was much higher in Malpighian tubules and the gut (43), implying that the bacteriome is not the best habitat for *Wolbachia*.

The uninucleate bacteriocytes were rich in host organelles including mitochondria, rough endoplasmic reticula, and the Golgi apparatus, many of which were observed surrounding and in contact with *Carsonella* cells (Fig. 2). This arrangement potentially facilitates the delivery of proteins and metabolites required to maintain the bacteriocyte symbiosis, being consistent with metabolic complementarity between the host and *Carsonella*, which was suggested by genomic and transcriptomic analyses (8, 16, 18 – 20, 36). In the case of aphids, sternorrhynchan insects closely related to psyllids, proteins synthesized by the host bacteriocytes are shown to be transported to the endosymbiont (62). It would be interesting to assess if similar integrated systems have evolved in Psylloidea. The close proximity between *Carsonella* and host organelles is reminiscent of the case of the protist *Paramecium bursaria*. Intimate connections were identified between the *P. bursaria* mitochondria and symbiotic algae via the host membrane that contains algae, implying the transfer of proteins and essential metabolites (75). In *P. bursaria*, only cryo-fixation identified direct contacts of the vacuole membrane to the mitochondrial membrane and the cell wall of the symbiotic algae, which was unattained by the chemical fixation. Therefore, cryo-electron microscopy may reveal finer structures showing more intimate host-symbiont interactions in the bacteriome symbiotic system of *D. citri*.

This study identified fiber bundles just under the envelope of *Carsonella* (Fig. 2C). The fibers were arranged both longitudinally and transversely, suggesting to form a net-like structure lining the *Carsonella* envelope. These structures appeared to correspond to the “peripheral macula” previously reported for *Carsonella* of other psyllid species, *C. pyricola* (Psyllidae: Psyllinae) (12) and *A. mori* (Psyllidae: Psyllinae) (13). However, to the best of our knowledge, similar structures have not been observed in any other bacteria, including other bacteriome-associated symbionts in insects (76 – 82). As *Carsonella* cells are relatively large and can be extremely long (9, 18, 21), these linings may play an important role in providing mechanical support to the cell structure of *Carsonella*. Within *Carsonella* cells, numerous ribosomes were observed, parts of which formed conspicuous aggregations (Fig. 2C and D) reminiscent of the nucleolus, the site of ribosome biogenesis in eukaryotes (83). A previous study showed that *Carsonella* stained with DAPI exhibits numerous spots with higher signal intensities within the cell (21). The aggregates observed in the present study may correspond to these signal spots, which potentially contain higher amounts of nucleic acids. Further studies determining the precise localization of genomic copies, ribosomes, and their precursors in *Carsonella* would provide clues to understand the mechanisms for organizing numerous copies (21) of the drastically reduced genome (8, 18 – 20) and expressing genes encoded therein.

A characteristic feature of *Profftella* cells revealed in this study is the presence of tubular structures, which are approximately 300 nm in diameter, consisting of several intertwined fibers (Fig. 3A through D). Our previous metagenomic analysis did not detect DNA sequences corresponding to known viruses or bacteria other than *Carsonella*, *Profftella*, or *Wolbachia* (8), suggesting that the tubes are not parasitic microbes residing in *Profftella*. A large part of ribosomes appeared to colocalize with the tubes (Fig. 3C and D), implying the involvement of these structures in the gene expression of *Profftella*. Further studies are required to determine the finer structures and functions of the tubes. This study showed that a three-layered envelope encapsulates *Profftella* cells, with the outermost layer probably derived from the host (Fig. 3F). The encasement of symbionts in a host membrane is a typical feature of various intracellular symbiotic systems (75 – 82), which would facilitate the host’s control over the symbionts. Apparent cell protrusions were observed on the surface of some *Profftella* cells (Fig. 3A, B, F, and G). These structures may facilitate interactions with the surrounding environment, including host organelles and cytoplasm of the syncytium, and other *Profftella* cells. Besides, structures that appeared to be degrading *Profftella* were occasionally observed in the syncytium (Fig. 3G through I). The degradation of *Profftella* would yield an opportunity to release the genomic DNA of *Profftella* into the cytoplasm of the syncytium. Although this study utilized *D. citri* uninfected with “*Candidatus* Liberibacter” spp., a previous study detected a considerable abundance of “*Candidatus* Liberibacter asiaticus” in the bacteriome of infected *D. citri* (43). Thus, the freed *Profftella* genome can potentially be a source of horizontal acquisition by the *Liberibacter* lineages, as exemplified in a transporter gene preserved in the Liberibacter lineages except for the most basal species *L. crescens* (64).

Host organelles, including mitochondria and endoplasmic reticula with ribosomes, were less abundant in the syncytium than in the uninucleate bacteriocytes (Fig. 3A through I). Moreover, mitochondria in the syncytium had fewer cristae than those in the uninucleate bacteriocytes (Fig. 3E). These observations imply that the syncytium is metabolically less active in comparison to bacteriocytes.

### Conclusions

This study revealed ultrastructures of the *D. citri* bacteriome, including the following: (i) fiber bundles forming a net-like structure lining the *Carsonella* envelope, which may be essential to support the large and long *Carsonella* cells, (ii) aggregates of ribosomes in *Carsonella*, which are reminiscent of the eukaryotic nucleolus, (iii) many host organelles in contact with *Carsonella* cells in the uninucleate bacteriocyte, which may facilitate the delivery of proteins and metabolites required to maintain the bacteriocyte symbiosis, (iv) numerous tubes consisting of several intertwined fibers in *Profftella*, the function of which is uncertain, (v) protrusions on the surface of *Profftella*, which may facilitate interactions with the host cell components and other *Profftella* cells, (vi) degrading *Profftella* in the syncytium, providing opportunities for horizontal gene transfer between symbionts, and (vii) less abundant organelles, including mitochondria with less developed cristae, in the syncytium than in the uninucleate bacteriocytes. These observations established a basis for understanding interactions among the host cells and bacterial symbionts in *D. citri*.

## References

[1] Moran NA , McCutcheon JP , Nakabachi A . 2008. Genomics and evolution of heritable bacterial symbionts. Annu Rev Genet 42:165–190. doi:10.1146/annurev.genet.41.110306.130119 18983256

[2] McCutcheon JP , Boyd BM , Dale C . 2019. The life of an insect endosymbiont from the cradle to the grave. Curr Biol 29:R485–R495. doi:10.1016/j.cub.2019.03.032 31163163

[3] Perreau J , Moran NA . 2022. Genetic innovations in animal–microbe symbioses. Nat Rev Genet 23:23–39. doi:10.1038/s41576-021-00395-z 34389828 PMC8832400

[4] Grafton-Cardwell EE , Stelinski LL , Stansly PA . 2013. Biology and management of Asian citrus psyllid, vector of the huanglongbing pathogens. Annu Rev Entomol 58:413–432. doi:10.1146/annurev-ento-120811-153542 23317046

[5] Hu B , Rao MJ , Deng X , Pandey SS , Hendrich C , Ding F , Wang N , Xu Q . 2021. Molecular signatures between citrus and Candidatus Liberibacter asiaticus. PLoS Pathog 17:e1010071. doi:10.1371/journal.ppat.1010071 34882744 PMC8659345

[6] Killiny N . 2022. Made for each other: vector-pathogen interfaces in the huanglongbing pathosystem. Phytopathology 112:26–43. doi:10.1094/PHYTO-05-21-0182-FI 34096774

[7] Subandiyah S , Nikoh N , Tsuyumu S , Somowiyarjo S , Fukatsu T . 2000. Complex endosymbiotic microbiota of the citrus psyllid Diaphorina citri (Homoptera: psylloidea). Zool Sci 17:983–989. doi:10.2108/zsj.17.983

[8] Nakabachi A , Ueoka R , Oshima K , Teta R , Mangoni A , Gurgui M , Oldham NJ , van Echten-Deckert G , Okamura K , Yamamoto K , Inoue H , Ohkuma M , Hongoh Y , Miyagishima S , Hattori M , Piel J , Fukatsu T . 2013. Defensive bacteriome symbiont with a drastically reduced genome. Curr Biol 23:1478–1484. doi:10.1016/j.cub.2013.06.027 23850282

[9] Dan H , Ikeda N , Fujikami M , Nakabachi A . 2017. Behavior of bacteriome symbionts during transovarial transmission and development of the Asian citrus psyllid. PLoS One 12:e0189779. doi:10.1371/journal.pone.0189779 29240843 PMC5730177

[10] Profft J . 1937. Beiträge zur Symbiose der Aphiden und Psylliden. Z Morph u Okol Tiere 32:289–326. doi:10.1007/BF00403077

[11] Buchner P . 1965. Endosymbiosis of animals with plant microorganisms. Interscience, New York.

[12] Chang KP , Musgrave AJ . 1969. Histochemistry and ultrastructure of the mycetome and its 'symbiotes' in the pear psylla, Psylla pyricola Foerster (Homoptera). Tissue Cell 1:597–606. doi:10.1016/s0040-8166(69)80034-0 18631487

[13] Waku Y , Endo Y . 1987. Ultrastructure and life cycle of the symbionts in a homopteran insect, Anomoneura mori schwartz (Psyllidae). Appl Entomol Zool 22:630–637. doi:10.1303/aez.22.630

[14] Fukatsu T , Nikoh N . 1998. Two intracellular symbiotic bacteria from the mulberry psyllid Anomoneura Mori (insecta, Homoptera). Appl Environ Microbiol 64:3599–3606. doi:10.1128/AEM.64.10.3599-3606.1998 9758773 PMC106470

[15] Nakabachi A , Koshikawa S , Miura T , Miyagishima S . 2010. Genome size of Pachypsylla venusta (Hemiptera: psyllidae) and the ploidy of its bacteriocyte, the symbiotic host cell that harbors intracellular mutualistic bacteria with the smallest cellular genome. Bull Entomol Res 100:27–33. doi:10.1017/S0007485309006737 19302725

[16] Sloan DB , Nakabachi A , Richards S , Qu J , Murali SC , Gibbs RA , Moran NA . 2014. Parallel histories of horizontal gene transfer facilitated extreme reduction of endosymbiont genomes in sap-feeding insects. Mol Biol Evol 31:857–871. doi:10.1093/molbev/msu004 24398322 PMC3969561

[17] Thao ML , Moran NA , Abbot P , Brennan EB , Burckhardt DH , Baumann P . 2000. Cospeciation of psyllids and their primary prokaryotic endosymbionts. Appl Environ Microbiol 66:2898–2905. doi:10.1128/AEM.66.7.2898-2905.2000 10877784 PMC92089

[18] Nakabachi A , Yamashita A , Toh H , Ishikawa H , Dunbar HE , Moran NA , Hattori M . 2006. The 160-kilobase genome of the bacterial endosymbiont Carsonella. Science 314:267. doi:10.1126/science.1134196 17038615

[19] Sloan DB , Moran NA . 2012. Genome reduction and co-evolution between the primary and secondary bacterial symbionts of psyllids. Mol Biol Evol 29:3781–3792. doi:10.1093/molbev/mss180 22821013 PMC3494270

[20] Nakabachi A , Piel J , Malenovský I , Hirose Y . 2020. Comparative genomics underlines multiple roles of Profftella, an obligate symbiont of psyllids: providing toxins, vitamins, and carotenoids. Genome Biol Evol 12:1975–1987. doi:10.1093/gbe/evaa175 32797185 PMC7643613

[21] Nakabachi A , Moran NA . 2022. Extreme polyploidy of Carsonella, an organelle-like bacterium with a drastically reduced genome. Microbiol Spectr 10:e0035022. doi:10.1128/spectrum.00350-22 35435757 PMC9241722

[22] Ziegler H , Pirson A , Zimmermann MH . 1975. Nature of transported substances, p 59–100. In Zimmermann MH , Milburn JA (ed), Transport in plants I. Springer-Verlag, New York.

[23] Sandström J , Moran N . 1999. How nutritionally imbalanced is phloem sap for aphids. Entomol Exp Appl 91:203–210. doi:10.1046/j.1570-7458.1999.00485.x

[24] Spaulding AW , von Dohlen CD . 2001. Psyllid endosymbionts exhibit patterns of co-speciation with hosts and destabilizing substitutions in ribosomal RNA. Insect Mol Biol 10:57–67. doi:10.1046/j.1365-2583.2001.00231.x 11240637

[25] Hall AAG , Morrow JL , Fromont C , Steinbauer MJ , Taylor GS , Johnson SN , Cook JM , Riegler M . 2016. Codivergence of the primary bacterial endosymbiont of psyllids versus host switches and replacement of their secondary bacterial endosymbionts. Environ Microbiol 18:2591–2603. doi:10.1111/1462-2920.13351 27114069

[26] Nakabachi A , Malenovský I , Gjonov I , Hirose Y . 2020. 16S rRNA sequencing detected Profftella, Liberibacter, Wolbachia, and Diplorickettsia from relatives of the Asian citrus psyllid. Microb Ecol 80:410–422. doi:10.1007/s00248-020-01491-z 32052099

[27] Nakabachi A , Inoue H , Hirose Y . 2022. Microbiome analyses of 12 psyllid species of the family Psyllidae identified various bacteria including Fukatsuia and Serratia symbiotica, known as secondary symbionts of aphids. BMC Microbiol 22:15. doi:10.1186/s12866-021-02429-2 34996376 PMC8740488

[28] Nakabachi A , Inoue H , Hirose Y . 2022. High-resolution microbiome analyses of nine psyllid species of the family Triozidae identified previously unrecognized but major bacterial populations, including Liberibacter and Wolbachia of supergroup O. Microbes Environ 37:ME22078. doi:10.1264/jsme2.ME22078 36476840 PMC9763047

[29] Maruyama J , Inoue H , Hirose Y , Nakabachi A . 2023. 16S rRNA gene sequencing of six psyllid species of the family Carsidaridae identified various bacteria including Symbiopectobacterium. Microbes Environ 38:ME23045. doi:10.1264/jsme2.ME23045 37612118 PMC10522848

[30] Morrow JL , Hall AAG , Riegler M . 2017. Symbionts in waiting: the dynamics of incipient endosymbiont complementation and replacement in minimal bacterial communities of psyllids. Microbiome 5:58. doi:10.1186/s40168-017-0276-4 28587661 PMC5461708

[31] Thao ML , Clark MA , Baumann L , Brennan EB , Moran NA , Baumann P . 2000. Secondary endosymbionts of psyllids have been acquired multiple times. Curr Microbiol 41:300–304. doi:10.1007/s002840010138 10977900

[32] Yamada T , Hamada M , Floreancig P , Nakabachi A . 2019. Diaphorin, a polyketide synthesized by an intracellular symbiont of the Asian citrus psyllid, is potentially harmful for biological control agents. PLoS One 14:e0216319. doi:10.1371/journal.pone.0216319 31048920 PMC6497295

[33] Nakabachi A , Okamura K . 2019. Diaphorin, a polyketide produced by a bacterial symbiont of the Asian citrus psyllid, kills various human cancer cells. PLoS One 14:e0218190. doi:10.1371/journal.pone.0218190 31181122 PMC6557518

[34] Nakabachi A , Fujikami M . 2019. Concentration and distribution of diaphorin, and expression of diaphorin synthesis genes during Asian citrus psyllid development. J Insect Physiol 118:103931. doi:10.1016/j.jinsphys.2019.103931 31442480

[35] Tanabe N , Takasu R , Hirose Y , Kamei Y , Kondo M , Nakabachi A . 2022. Diaphorin, a polyketide produced by a bacterial symbiont of the Asian citrus psyllid, inhibits the growth and cell division of Bacillus subtilis but promotes the growth and metabolic activity of Escherichia coli. Microbiol Spectr 10:e0175722. doi:10.1128/spectrum.01757-22 35894614 PMC9430481

[36] Kwak Y , Hansen AK . 2023. Unveiling metabolic integration in psyllids and their nutritional endosymbionts through comparative transcriptomics analysis. iScience 26:107930. doi:10.1016/j.isci.2023.107930 37810228 PMC10558732

[37] Saha S , Hunter WB , Reese J , Morgan JK , Marutani-Hert M , Huang H , Lindeberg M , Zilberstein D . 2012. Survey of endosymbionts in the Diaphorina citri metagenome and assembly of a Wolbachia wDi draft genome. PLoS ONE 7:e50067. doi:10.1371/journal.pone.0050067 23166822 PMC3500351

[38] Guidolin AS , Cônsoli FL . 2013. Molecular characterization of Wolbachia strains associated with the invasive Asian citrus psyllid Diaphorina citri in Brazil. Microb Ecol 65:475–486. doi:10.1007/s00248-012-0150-7 23269454

[39] Dossi FCA , da Silva EP , Cônsoli FL . 2014. Population dynamics and growth rates of endosymbionts during Diaphorina citri (Hemiptera, Liviidae) ontogeny. Microb Ecol 68:881–889. doi:10.1007/s00248-014-0463-9 25037159

[40] Lashkari M , Manzari S , Sahragard A , Malagnini V , Boykin LM , Hosseini R . 2014. Global genetic variation in the Asian citrus psyllid, Diaphorina citri (Hemiptera: Liviidae) and the endosymbiont Wolbachia: links between Iran and the USA detected. Pest Manag Sci 70:1033–1040. doi:10.1002/ps.3643 24002991

[41] Chu C-C , Gill TA , Hoffmann M , Pelz-Stelinski KS . 2016. Inter-population variability of endosymbiont densities in the Asian citrus psyllid (Diaphorina Citri Kuwayama). Microb Ecol 71:999–1007. doi:10.1007/s00248-016-0733-9 26846216 PMC4944574

[42] Meng L , Li X , Cheng X , Zhang H . 2019. 16S rRNA gene sequencing reveals a shift in the microbiota of Diaphorina Citri during the psyllid life cycle. Front Microbiol 10:1948. doi:10.3389/fmicb.2019.01948 31507561 PMC6716071

[43] Hosseinzadeh S , Shams-Bakhsh M , Mann M , Fattah-Hosseini S , Bagheri A , Mehrabadi M , Heck M . 2019. Distribution and variation of bacterial endosymbiont and "Candidatus Liberibacter asiaticus" titer in the huanglongbing insect vector, Diaphorina citri Kuwayama. Microb Ecol 78:206–222. doi:10.1007/s00248-018-1290-1 30474731

[44] Chu C-C , Hoffmann M , Braswell WE , Pelz-Stelinski KS . 2019. Genetic variation and potential coinfection of Wolbachia among widespread Asian citrus psyllid (Diaphorina citri Kuwayama) populations. Insect Sci 26:671–682. doi:10.1111/1744-7917.12566 29286204 PMC7379232

[45] Nakabachi A , Ishikawa H . 1997. Differential display of mRNAs related to amino acid metabolism in the endosymbiotic system of aphids. Insect Biochem Mol Biol 27:1057–1062. doi:10.1016/s0965-1748(97)00092-1 9569646

[46] Kikuchi Y . 2009. Endosymbiotic bacteria in insects: their diversity and culturability. Microbes Environ 24:195–204. doi:10.1264/jsme2.me09140s 21566374

[47] Uchi N , Fukudome M , Nozaki N , Suzuki M , Osuki K-I , Shigenobu S , Uchiumi T . 2019. Antimicrobial activities of cysteine-rich peptides specific to bacteriocytes of the pea aphid Acyrthosiphon pisum. Microbes Environ 34:155–160. doi:10.1264/jsme2.ME18148 30905896 PMC6594739

[48] Gerardo NM , Altincicek B , Anselme C , Barribeau SM , Duncan EJ , Latorre A , Rahbé Y . 2010. Immunity and other defenses in pea aphids, Acyrthosiphon pisum. Genome Biol 11:R21 11. doi:10.1186/gb-2010-11-2-r21 PMC2872881 20178569

[49] Nikoh N , McCutcheon JP , Kudo T , Miyagishima S , Moran NA , Nakabachi A . 2010. Bacterial genes in the aphid genome: absence of functional gene transfer from Buchnera to its host. PLoS Genet 6:e1000827. doi:10.1371/journal.pgen.1000827 20195500 PMC2829048

[50] Nakabachi A , Shigenobu S , Miyagishima S . 2010. Chitinase-like proteins encoded in the genome of the pea aphid, Acyrthosiphon pisum. Insect Mol Biol 19 Suppl 2:175–185. doi:10.1111/j.1365-2583.2009.00985.x 20482649

[51] Ramsey JS , MacDonald SJ , Jander G , Nakabachi A , Thomas GH , Douglas AE . 2010. Genomic evidence for complementary purine metabolism in the pea aphid, Acyrthosiphon pisum, and its symbiotic bacterium Buchnera aphidicola. Insect Mol Biol 19 Suppl 2:241–248. doi:10.1111/j.1365-2583.2009.00945.x 20482654

[52] Nakabachi A , Ishikawa H . 2001. Expression of host S-adenosylmethionine decarboxylase gene and polyamine composition in aphid bacteriocytes. Insect Biochem Mol Biol 31:491–496. doi:10.1016/s0965-1748(00)00156-9 11222959

[53] Nakabachi A , Ishikawa H . 2000. Polyamine composition and expression of genes related to polyamine biosynthesis in an aphid endosymbiont, Buchnera. Appl Environ Microbiol 66:3305–3309. doi:10.1128/AEM.66.8.3305-3309.2000 10919785 PMC92149

[54] International Aphid Genomics consortium . 2010. Genome sequence of the pea aphid Acyrthosiphon pisum. PLoS Biol 10.1371/journal.pbio.1000313 PMC2826372 20186266

[55] Nakabachi A , Miyagishima S . 2010. Expansion of genes encoding a novel type of dynamin in the genome of the pea aphid, Acyrthosiphon pisum. Insect Mol Biol 19 Suppl 2:165–173. doi:10.1111/j.1365-2583.2009.00941.x 20482648

[56] Nakabachi A , Ishikawa H . 1999. Provision of riboflavin to the host aphid, Acyrthosiphon pisum, by endosymbiotic bacteria, Buchnera. J Insect Physiol 45:1–6. doi:10.1016/s0022-1910(98)00104-8 12770389

[57] Nakabachi A , Ishikawa H , Kudo T . 2003. Extraordinary proliferation of microorganisms in aposymbiotic pea aphids, Acyrthosiphon pisum. J Invertebr Pathol 82:152–161. doi:10.1016/s0022-2011(03)00020-x 12676551

[58] Nakabachi A , Shigenobu S , Sakazume N , Shiraki T , Hayashizaki Y , Carninci P , Ishikawa H , Kudo T , Fukatsu T . 2005. Transcriptome analysis of the aphid bacteriocyte, the symbiotic host cell that harbors an endocellular mutualistic bacterium, Buchnera. Proc Natl Acad Sci U S A 102:5477–5482. doi:10.1073/pnas.0409034102 15800043 PMC555734

[59] Nikoh N , Nakabachi A . 2009. Aphids acquired symbiotic genes via lateral gene transfer. BMC Biol 7:12. doi:10.1186/1741-7007-7-12 19284544 PMC2662799

[60] Shigenobu S , Richards S , Cree AG , Morioka M , Fukatsu T , Kudo T , Miyagishima S , Gibbs RAA , Stern DLL , Nakabachi A . 2010. A full-length cDNA resource for the pea aphid, Acyrthosiphon pisum. Insect Mol Biol 19 Suppl 2:23–31. doi:10.1111/j.1365-2583.2009.00946.x 20482637 PMC4370113

[61] Tamborindeguy C , Monsion B , Brault V , Hunnicutt L , Ju HJ , Nakabachi A , Van Fleet E . 2010. A genomic analysis of transcytosis in the pea aphid, Acyrthosiphon pisum, a mechanism involved in virus transmission. Insect Mol Biol 19 Suppl 2:259–272. doi:10.1111/j.1365-2583.2009.00956.x 20482656

[62] Nakabachi A , Ishida K , Hongoh Y , Ohkuma M , Miyagishima S-Y . 2014. Aphid gene of bacterial origin encodes a protein transported to an obligate endosymbiont. Curr Biol 24:R640–R641. doi:10.1016/j.cub.2014.06.038 25050957

[63] Nakabachi A . 2015. Horizontal gene transfers in insects. Curr Opin Insect Sci 7:24–29. doi:10.1016/j.cois.2015.03.006 32131363

[64] Nakabachi A , Nikoh N , Oshima K , Inoue H , Ohkuma M , Hongoh Y , Miyagishima S , Hattori M , Fukatsu T . 2013. Horizontal gene acquisition of Liberibacter plant pathogens from a bacteriome-confined endosymbiont of their psyllid vector. PLoS One 8:e82612. doi:10.1371/journal.pone.0082612 24349319 PMC3857777

[65] Jain M , Fleites LA , Gabriel DW , McMahon K , Lindow S , Hall D . 2017. A small Wolbachia protein directly represses phage lytic cycle genes in “Candidatus Liberibacter asiaticus” within psyllids. mSphere 2. doi:10.1128/mSphereDirect.00171-17 PMC5463029 28608866

[66] Kruse A , Fattah-Hosseini S , Saha S , Johnson R , Warwick E , Sturgeon K , Mueller L , MacCoss MJ , Shatters RG , Cilia Heck M , Pappu HR . 2017. Combining ’omics and microscopy to visualize interactions between the Asian citrus psyllid vector and the Huanglongbing pathogen Candidatus Liberibacter asiaticus in the insect gut. PLoS ONE 12:e0179531. doi:10.1371/journal.pone.0179531 28632769 PMC5478155

[67] Ammar E-D , Hall DG , Shatters RG . 2017. Ultrastructure of the salivary glands, alimentary canal and bacteria-like organisms in the Asian citrus psyllid, vector of citrus huanglongbing disease bacteria. J Microsc Ultrastruct 5:9–20. doi:10.1016/j.jmau.2016.01.005 30023232 PMC6014262

[68] Ammar E-D , Achor D , Levy A . 2019. Immuno-ultrastructural localization and putative multiplication sites of huanglongbing bacterium in Asian citrus psyllid Diaphorina citri. Insects 10:422. doi:10.3390/insects10120422 31771154 PMC6955907

[69] Sato T . 1968. A modified method for lead staining of thin sections. J Electron Microsc (Tokyo) 17:158–159. 4177281

[70] Li S-J , Ahmed MZ , Lv N , Shi P-Q , Wang X-M , Huang J-L , Qiu B-L . 2017. Plantmediated horizontal transmission of Wolbachia between whiteflies. ISME J 11:1019–1028. doi:10.1038/ismej.2016.164 27935594 PMC5364347

[71] Balaji S , Jayachandran S , Prabagaran SR . 2019. Evidence for the natural occurrence of Wolbachia in aedes aegypti mosquitoes. FEMS Microbiol Lett 366:fnz055. doi:10.1093/femsle/fnz055 30869785

[72] McMeniman CJ , Lane AM , Fong AWC , Voronin DA , Iturbe-Ormaetxe I , Yamada R , McGraw EA , O’Neill SL . 2008. Host adaptation of a Wolbachia strain after long-term serial passage in mosquito cell lines. Appl Environ Microbiol 74:6963–6969. doi:10.1128/AEM.01038-08 18836024 PMC2583474

[73] White PM , Pietri JE , Debec A , Russell S , Patel B , Sullivan W . 2017. Mechanisms of horizontal cell-to-cell transfer of Wolbachia spp. in Drosophila melanogaster. Appl Environ Microbiol 83:e03425-16. doi:10.1128/AEM.03425-16 28087534 PMC5359480

[74] Dittmer J , Beltran-Bech S , Lesobre J , Raimond M , Johnson M , Bouchon D . 2014. Host tissues as microhabitats for Wolbachia and quantitative insights into the bacterial community in terrestrial Isopods. Mol Ecol 23:2619–2635. doi:10.1111/mec.12760 24750488

[75] Song C , Murata K , Suzaki T . 2017. Intracellular symbiosis of algae with possible involvement of mitochondrial dynamics. Sci Rep 7:1221. doi:10.1038/s41598-017-01331-0 28450706 PMC5430747

[76] Griffiths GW , Beck SD . 1973. Intracellular symbiotes of the pea aphid, Acyrthosiphon pisum. J Insect Physiol 19:75–84. doi:10.1016/0022-1910(73)90223-0 4364847

[77] Griffiths GW , Beck SD . 1975. Ultrastructure of pea aphid mycetocytes: evidence for symbiote secretion. Cell Tissue Res 159:351–367. doi:10.1007/BF00221782 807329

[78] Houk EJ , Griffiths GW , Hadjokas NE , Beck SD . 1977. Peptidoglycan in the cell wall of the primary intracellular symbiote of the pea aphid. Science 198:401–403. doi:10.1126/science.198.4315.401 17809442

[79] Aksoy S . 1995. Wigglesworthia gen. nov. and Wigglesworthia glossinidia sp. nov., taxa consisting of the mycetocyte-associated, primary endosymbionts of tsetse flies. Int J Syst Bacteriol 45:848–851. doi:10.1099/00207713-45-4-848 7547309

[80] von Dohlen CD , Kohler S , Alsop ST , McManus WR . 2001. Mealybug beta-proteobacterial endosymbionts contain gamma-proteobacterial symbionts. Nature 412:433–436. doi:10.1038/35086563 11473316

[81] Matsuura Y , Kikuchi Y , Hosokawa T , Koga R , Meng X-Y , Kamagata Y , Nikoh N , Fukatsu T . 2012. Evolution of symbiotic organs and endosymbionts in lygaeid stinkbugs. ISME J 6:397–409. doi:10.1038/ismej.2011.103 21814289 PMC3260496

[82] Santos-Garcia D , Silva FJ , Moya A , Latorre A . 2014. No exception to the rule: Candidatus Portiera aleyrodidarum cell wall revisited. FEMS Microbiol Lett 360:132–136. doi:10.1111/1574-6968.12595 25196985

[83] Boisvert F-M , van Koningsbruggen S , Navascués J , Lamond AI . 2007. The multifunctional nucleolus. Nat Rev Mol Cell Biol 8:574–585. doi:10.1038/nrm2184 17519961

